# Susceptibility of epithelial cells cultured from different regions of human cervix to HPV16-induced immortalization

**DOI:** 10.1371/journal.pone.0199761

**Published:** 2018-06-26

**Authors:** Han Deng, Eric Hillpot, Philomina Yeboah, Sumona Mondal, Craig D. Woodworth

**Affiliations:** 1 Department of Biology, Clarkson University, Potsdam, New York, United States of America; 2 Department of Mathematics, Clarkson University, Potsdam, New York, United States of America; Georgetown University, UNITED STATES

## Abstract

Persistent infection with high-risk human papillomavirus (HPV) is a major risk factor for cervical cancer. Greater than 90% of these cancers originate in the cervical transformation zone (TZ), a narrow region of metaplastic squamous epithelium that develops at the squamocolumnar junction between the ectocervix and endocervix. It is unclear why the TZ has high susceptibility to malignant transformation and few studies have specifically examined cells from this region. We hypothesized that cells cultured from TZ are more susceptible to cellular immortalization, an alteration that contributes to malignant development. We cultured primary epithelial cells from each region of human cervix (ectocervix, endocervix and TZ) and measured susceptibility to immortalization after transfection with the complete HPV-16 genome or infection of HPV16 E6/E7 retroviruses. Cells cultured from each cervical region expressed keratin markers (keratin 14 and 18) that confirmed their region of origin. In contrast to our prediction, cells from TZ were equally susceptible to immortalization as cells from ectocervix or endocervix. Thus, increased susceptibility of the TZ to cervical carcinogenesis is not due to increased frequency of immortalization by HPV-16. We developed a series of HPV16-immortalized cell lines from ectocervix, endocervix and TZ that will enable comparisons of how these cells respond to factors that promote cervical carcinogenesis.

## Introduction

Cervical cancer is a leading cause of cancer death in women worldwide [1] and persistent infection with high-risk HPV types such as HPV16 is the major risk factor for this disease [2,3]. The HPV E6 and E7 oncogenes are selectively retained and expressed in almost all cervical cancers. High-risk HPV16 E6 and E7 genes are sufficient to immortalize human cervical epithelial cells [4] and cell immortalization is an important early step in malignant development [5]. Although infection with high-risk HPV types is necessary for cervical cancer, it is not sufficient. HPV infections occur frequently in sexually active women, but most are recognized by the immune system and eliminated [6]. It is unclear why some high-risk HPV infections progress to cancer while many others do not.

**Fig 1:**
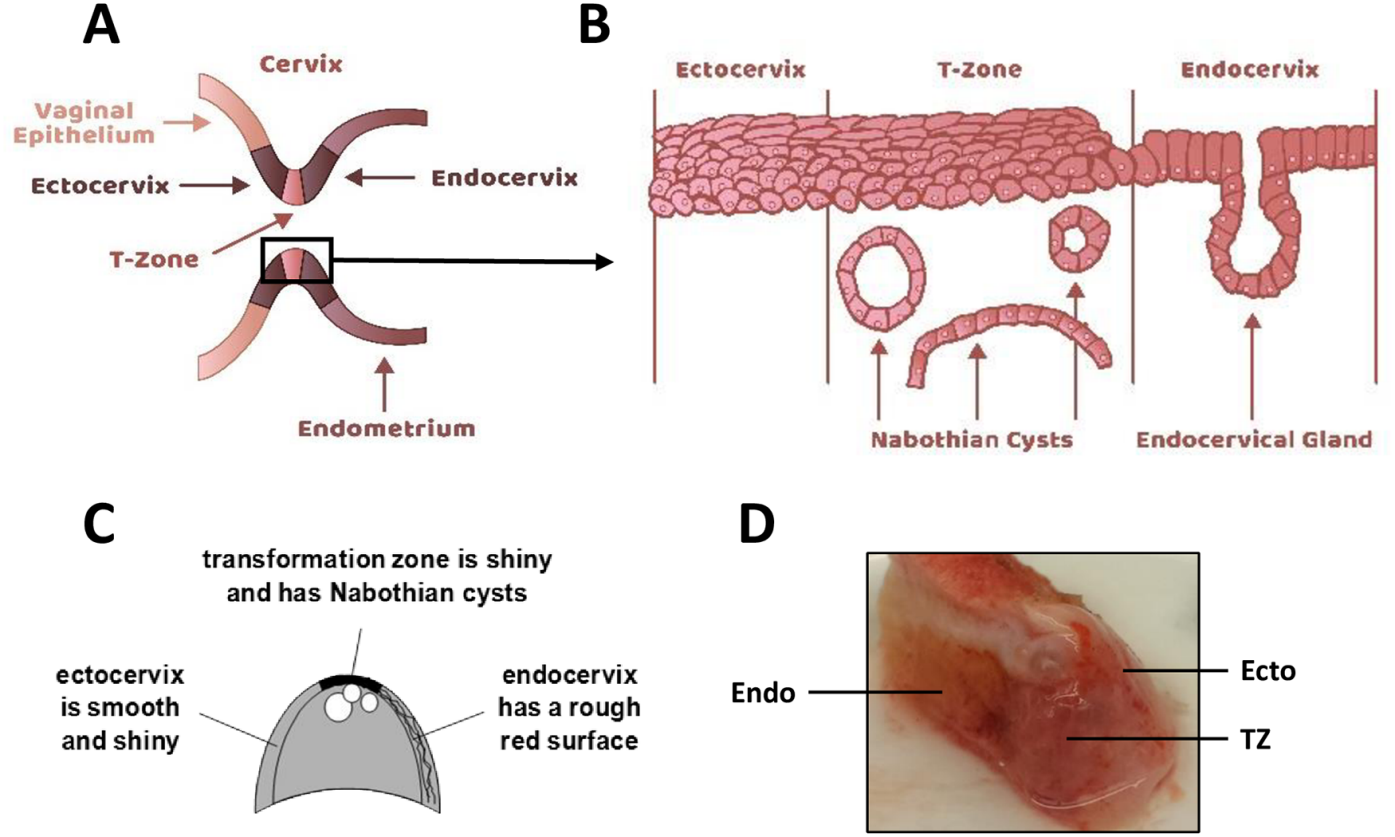
Structure and histology of the cervical TZ. A. Schematic representation of the cervix showing the TZ between ectocervix and endocervix. B. Histology of the cervical TZ showing the stratified squamous epithelium and underlying Nabothian cysts. C. Schematic showing the surface features of ectocervix, endocervix and TZ that aid in tissue dissection. The ectocervix is easily identified because the surface is smooth, white, and shiny with no mucous. The endocervix surface is rough, red in color, and covered with mucous. The TZ contains Nabothian cysts (swollen glands due to occlusion of ducts by squamous metaplasia). These large cysts are easily visible and diagnostic for the TZ. D. Photograph of a typical cervical specimen showing each region.

Although high-risk HPV infections occur throughout the cervix and vagina [7], about 90% of cervical cancers develop within a small anatomic region [8] known as the cervical transformation zone (TZ). This region lies between the stratified squamous epithelium of the ectocervix and the columnar epithelium of the endocervix (Fig 1). The TZ is composed of metaplastic squamous cells derived from stem cells (reserve cells) of the endocervix. Although the majority of cervical cancers originate from the TZ, it is unclear why this region is so susceptible to malignant conversion. Several hypotheses have been suggested including the existence of localized immune suppression in this region [9], increased expression of estrogen receptors on metaplastic epithelial or stromal cells [10], increased cell proliferation and unstable differentiation of metaplastic cells [11], or an increased concentration of stem cells within the TZ [12]. There has been limited research on cells from TZ to understand their increased risk of carcinogenic progression. We examined the hypothesis that epithelial cells cultured from the TZ are more susceptible to immortalization by high-risk HPV16 than are cells of the surrounding ectocervix or endocervix. We used three different immortalization assays with the complete HPV16 genome or retroviruses encoding HPV16 E6 and E7 oncogenes. In contrast to our prediction, we found that TZ cells were equally susceptible to immortalization by HPV16 as cells from ectocervix or endocervix.

## Materials and methods

### Cell culture

Samples of human cervical tissue were purchased from the Co-operative Human Tissue Network and shipped overnight on wet ice. Tissues had no patient identification and all specimens were originally procured for other purposes. Thus, our experiments were exempted from Institutional Review Board approval of Human Subjects Research by Clarkson University. Human epithelial cells were isolated from fresh tissue as previously described [13] using dispase to separate epithelium from dermis and trypsin to digest aggregates of cells into small cell clusters and individual cells. Cell cultures were maintained in Keratinocyte Serum-Free Medium (KSFM, GIBCO) containing antibiotics. In some experiments, cells from TZ and endocervix were cultured in EpiLife medium (GIBCO). In selected experiments, primary cervical cultures were checked for the presence of HPV16 DNA using E6 and E7 primers. No evidence of HPV infection was found and no spontaneous immortalization occurred in any transfection experiment. Primary or secondary cultures were used for immortalization assays. Retrovirus producer cell lines were maintained in Dulbecco’s Minimal Essential Medium (DMEM) containing 10% fetal bovine serum (FBS) plus antibiotics. Human stromal cells were isolated by collagenase digestion of cervical stromal tissue and cultured in DMEM with 10% FBS. Co-cultures of stromal and cervical epithelial cells were performed as described [14].

### Transfection efficiency

Cervical cells were transfected with β-galactosidase reporter gene DNA using lipofectamine 3000 reagent (Invitrogen). After 24 h, transfected cells were stained blue by the β-gal staining kit (Invitrogen). Transfection efficiency was calculated by dividing the stained cell number by the total cell number.

### Immortalization assays

For HPV16 and HPV18 complete genome assays, secondary cultures of cervical cells were transfected with pMHPV16d or pSHPV18m plasmid DNAs [15] using lipofectamine 3000 reagent. After 24 h, cells were selected in 100 μg/ml G418 for 2 days. Three 60 mm dishes of cells from each cervical region were transfected and the number of cell dishes with immortal colonies was counted after negative control cultures (only the neomycin gene) became senescent. Simian Virus 40 (SV40) plasmid DNA pBRWT2 [16] containing the complete SV40 genome was used as a positive control for immortalization. In total, transfection experiments were performed on cervical cells isolated from 24 different patients (S1 Table). For retrovirus immortalization assays, cell cultures were infected with HPV16 E6/E7 retroviruses [17] as described [18] and selected in medium with 400 μg/ml G418 for 2 days. In total, retrovirus experiments were performed on cervical cells isolated from eight different patients (S2 Table). The same retrovirus stocks were used for all transfections from the same patient’s cervical tissue set. For co-culture assays, we cultured mitomycin C (MMC)-treated human cervical stromal cells with primary cervical epithelial cells. Epithelial cells were transfected with pMHPV16d plasmid containing the complete HPV16 genome using the same methods described in the immortalization assay performed in KSFM except that co-cultures were maintained in F12/DMEM raft medium containing 10% FBS [19]. For co-culture assays, stromal cells were first cultured in DMEM containing 10% FBS. Stromal cells were treated with 10μg/ml MMC for three hours then were washed twice with fresh medium. MMC-treated stromal cells were maintained two more days to make sure that they were quiescent but viable. MMC-treated cells were used at 80% confluence to allow epithelial cells to attach in the empty spaces. Prior to co-culture, epithelial cells were transfected with pMHPV16d plasmid containing the complete HPV16 genome. After G418 transfection, transfected epithelial cells were seeded to the stromal cell dishes and were fed with F12/DMEM raft medium containing 5% FBS. Epithelial cells grew in small colonies between stromal cells. When the diameter of colonies reached 1–2 cm, epithelial cells were subcultured in a new MMC-treated stromal cell dish. The co-culture immortalization assays were performed for eight weeks to judge whether cells were immortalized. In total, transfection experiments using co-cultures were performed on cells from five different patients.

### Immunofluorescence staining

Cells were cultured in four well TEK slides (Fisher) and fixed for 10 min using freshly made 4% neutral-buffered formalin. Primary rabbit monoclonal antibodies to keratin 14 (K14, ab51054, Abcam) and mouse monoclonal antibody to K18 (ab7797, Abcam) were used for double staining. Secondary donkey anti mouse antibody (ab150105, Abcam) with Alexa 488 tag and secondary goat anti rabbit antibody (ab150080, Abcam) with an Alexa 594 tag were used for visualization. Negative controls consisted of irrelevant primary antibodies including rabbit monoclonal isotype control (ab172730, Abcam) and mouse monoclonal isotype control (ab170190, Abcam). Primary mouse monoclonal antibody to K17 (ab188859, Abcam), mouse monoclonal antibody to MMP7 (sc-515703, Santa Cruz) and rabbit monoclonal antibodies to p63 (#13109S, Cell Signaling) were used for single staining. The dilution ratio for all primary antibodies was 1:300 and the dilution ratio for all secondary antibodies was 1:500. The gain and exposure time of the fluorescence microscope were adjusted to show positively stained cells while negative control cells had no staining. Staining percentage was calculated by dividing the positively stained cell number by the total cell number. Staining intensity was measured by ImageJ and the negative control was used to subtract background.

### Characterization of cell lines

Each of the 24 cell lines that we derived was tested by the commercial short tandem repeat (STR) profiling service at the American Type Culture Collection. Expression of HPV16 E6 and E7 RNAs were assessed by reverse transcription PCR using the specific oligonucleotide primers (HPV16 E6 forward 5'-GACCCAGAAAGTTACCACAG-3', and reverse 5'- GCAACAAGACATACATCGAC-3'; HPV16 E7 forward 5'-ATGACAGCTCAGAGGAGGAG-3', and reverse 5'- TCATAGTGTGCCCATTAACAG-3'). The resistance to terminal differentiation of each cell line was determined by plating cells at clonal density (50 cells / well of 6-well plate) and assessing growth in KSFM that lacked epidermal growth factor and bovine pituitary extract and was supplemented with 1.4 mM calcium chloride.

### Statistical analysis

Statistical analysis was performed using one-way analysis of variance (ANOVA) and Tukey's multiple comparison test for pairwise comparisons with IBM SPSS software version 24. A calculation of two-tailed p value less than 0.05 was considered significantly different.

## Results

### Characterization of cultured cells and confirmation of origin

**Fig 2:**
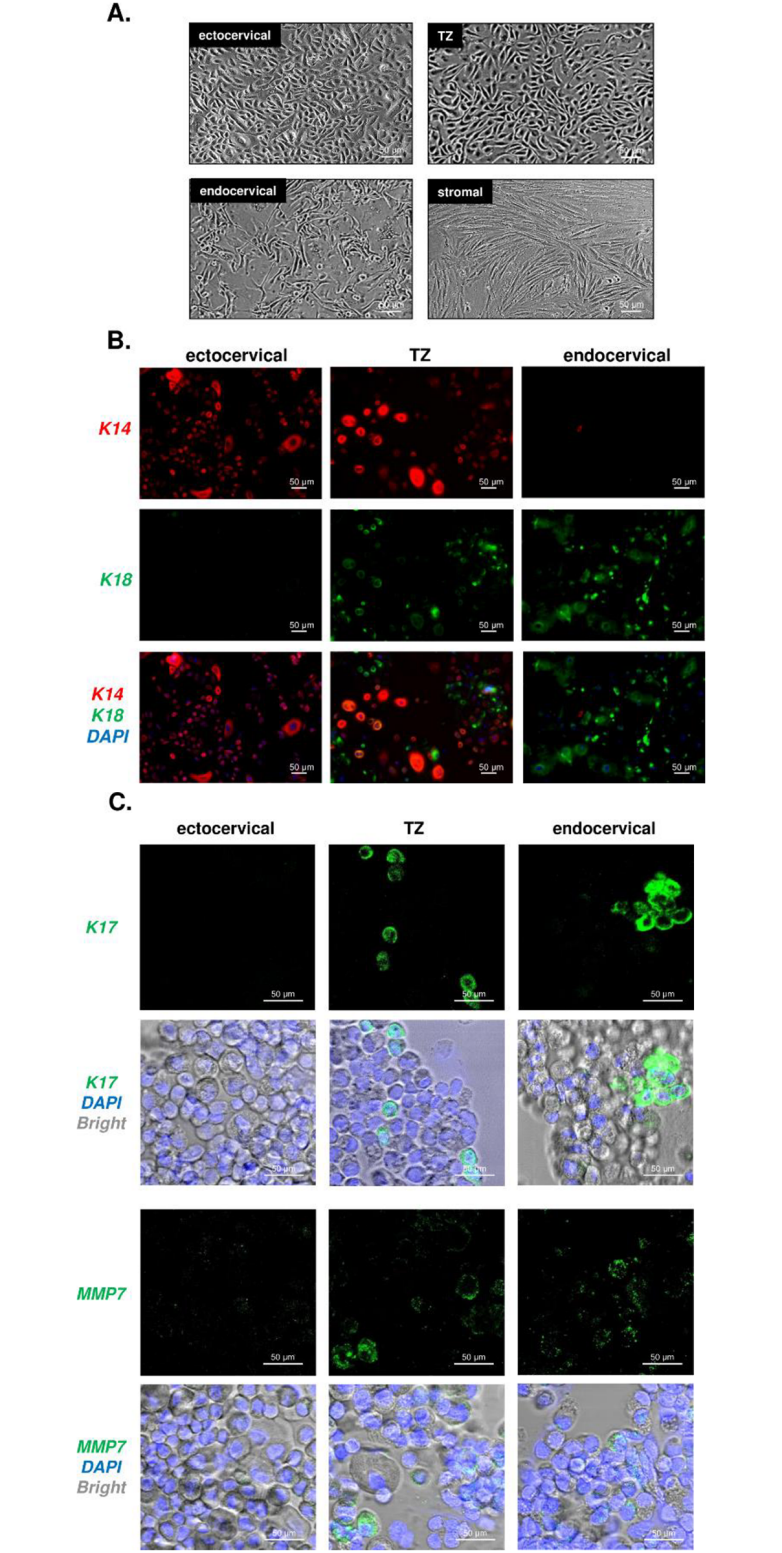
Cell morphology and keratin expression of monolayer cervical cell cultures. A. Phase contrast microscopy of primary human cervical cells from each region showing different cell morphology. B. K14 and K18 immunostaining showing different expression in cell culture *in vitro*. C. K17 and MMP7 immunostaining of cells cultured from each region of cervix. Irrelevant primary antibodies were used as the negative control for immunostaining.

We described procedures for isolation, culture, and characterization of human epithelial cells from ectocervix, endocervix and TZ [13,15]. Cells from each region can be cultured for 2 to 4 passages before senescence, and each exhibits a distinct morphology when maintained in keratinocyte serum-free medium (KSFM) in monolayer culture (Fig 2A). Cultures derived from endocervix are heterogeneous. Some cells proliferate whereas others contain vesicles and undergo secretory differentiation. Cultures from ectocervix are homogeneous and contain small epithelial cells that are motile and migrate throughout the dish. Cultures from the TZ have a unique morphology and growth pattern. They vary in shape and grow as colonies containing non-motile, closely associated cells. Stromal cells had a fibroblastic and elongated shape that differed from epithelial cells.

Different regions of cervical epithelium exhibit characteristic patterns of keratin gene expression *in vivo* [20]. Ectocervix expresses K14 but not K18, endocervix produces K18 but not K14, and TZ expresses both keratins. Putative stem cells of cervical epithelia (reserve cells) have been reported to contain K17 and transformation related protein p63 [20]. Matrix metalloproteinase 7 (MMP7) has been reported to be a marker for a discrete population of cells at the squamocolumnar junction that contributes to cervical cancer [21]. To confirm the origin of our epithelial cultures, freshly isolated cells from each cervical region were stained for K14 and K18 (Fig 2B) or either K17 or MMP7 (Fig 2C) using immunofluorescence.

**Fig 3:**
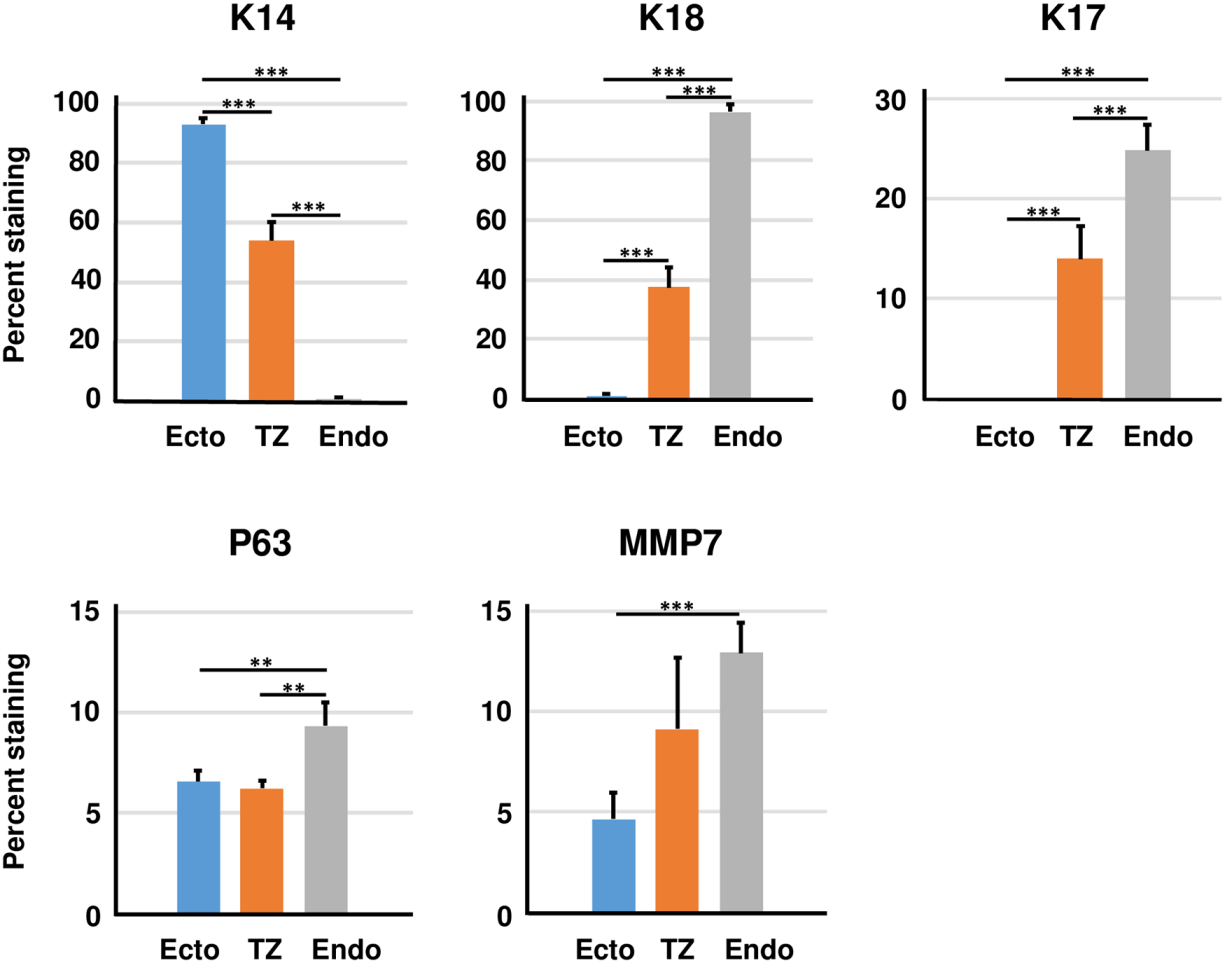
Quantitative analysis of cell marker expression by epithelial cells from each cervical region. Freshly isolated cells from each cervical region were stained with antibodies against K14, K18, K17, p63 or MMP7. Bars represent the mean ± standard error of three experiments using samples from different donors. The asterisks indicate statistical differences [** P < 0.01;*** P < 0.001].

We counted the percentage of epithelial cells that stained positively for each keratin marker (Fig 3). Greater than 90% of epithelial cells cultured from ectocervix expressed K14 but not K18, whereas over 90% of endocervical cells produced K18 but not K14. Most epithelial cells from TZ expressed either K14 or K18, but a small percentage produced both keratins (about 10%). A small percentage of cells cultured from endocervix and TZ expressed two stem cell markers, K17 and p63, but cells from ectocervix did not produce K17. We observed MMP7 expression in a small percentage of cells from each cervical region, but this marker was not specific for cells cultured from TZ. Overall, cervical epithelial cells isolated from each cervical region retained their characteristic keratin markers, confirming that they came from the corresponding ectocervical, TZ and endocervical regions.

### Immortalization of cells from TZ, ectocervix or endocervix

The major aim of this work was to compare the susceptibility of epithelial cells cultured from each cervical region to immortalization by HPV16, the most frequent high-risk HPV type. We examined three different immortalization assays. The first two were performed in a keratinocyte serum-free medium (KSFM) and consisted of (1) transfection with the complete HPV16 or HPV18 genome or (2) infection with high-titer retroviruses encoding the HPV16 E6 and E7 oncogenes. We also examined immortalization in a third assay that involved co-culture of HPV16-transfected cells with cervical stromal cells in serum-containing medium [14].

### Immortalization by the complete HPV16 genome

Secondary cultures of cells from each cervical region were maintained in KSFM and transfected with the plasmid pMHPV16d [15] which contains two copies of the complete HPV16 genome plus the neomycin resistance gene. Cells transfected with only the neomycin resistance gene served as a negative control and cells transfected with SV40 DNA served as positive control for immortalization. We examined 24 cervical tissues from different patients, and for each patient we transfected 3 x 60 mm culture dishes from each cervical region with HPV16 DNA (S1 Table). We briefly selected cultures in KSFM containing G418 to kill non-transfected cells, and then sub-cultured cells until the negative controls (neo only) became senescent. The HPV16 transfected cells that escaped senescence could be subcultured repeatedly to derive HPV16-immortalized cell lines (described below).

**Fig 4:**
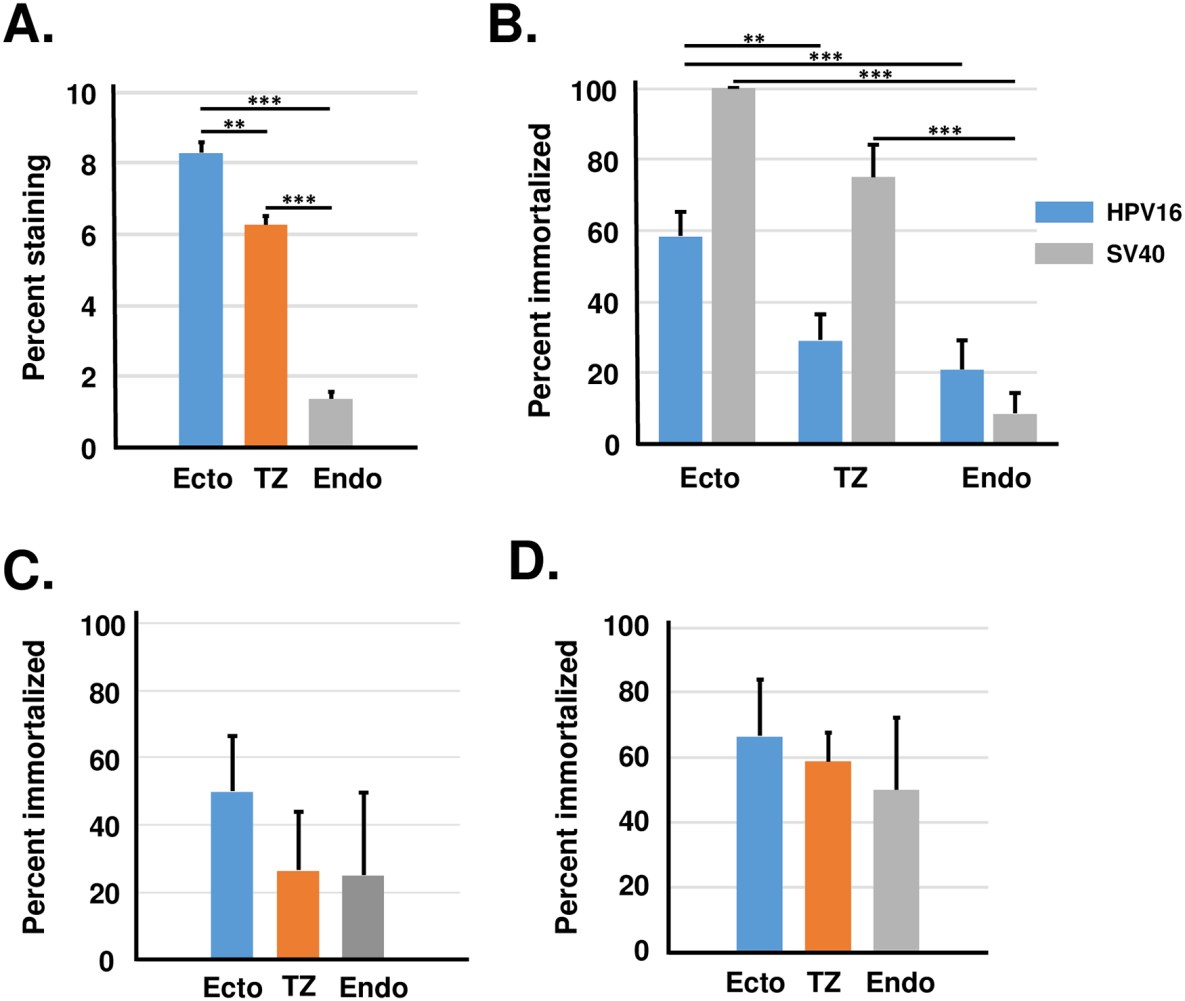
Transfection efficiency and immortalization efficiency after transfection with HPV16, HPV18 or SV40 DNA. A. Transfection efficiency of beta-galactosidase reporter gene in early passage cells from ectocervix, TZ and endocervix. The bars represent the mean ± standard error of 13 experiments using samples from different donors. B. Immortalization efficiency of cervical cells from each region after normalization for differences in transfection rate. Each experiment had negative controls consisting of cultures transfected with only the neomycin gene, and all negative control cultures became senescent after 2 to 3 passages. The bars represent the mean ± standard error of experiments from 24 donors (HPV16) or 12 donors (SV40). C. Immortalization efficiency of cervical cells by HPV18 after normalization for differences in transfection rate. The bars represent the mean ± standard error of experiments from five donors (HPV18). D. Immortalization efficiency of cervical cells by HPV16 when cells were maintained on collagen-coated cell culture plates. The asterisks indicate statistical differences [** P < 0.01; *** P < 0.001].

One potential problem was that cells cultured from each region differed in susceptibility to transfection (Fig 4A). Ectocervical cells were most easily transfected whereas endocervical cells were difficult to transfect. Because differences in the transfection efficiency could influence immortalization efficiency, we normalized each immortalization experiment for transfection efficiency using replicate cultures that were transfected with the reporter gene β-galactosidase. When data was normalized to control for transfection efficiency, we observed that ectocervical cells were immortalized slightly more frequently than either TZ or endocervical cells (Fig 4B). In immortalization assays using HPV18, we did not observe a significant difference between cervical cells from each region (Fig 4C). In ectocervical cultures, when immortalization did not occur, cells became senescent and their morphology resembled the non-transfected negative controls. In contrast, some TZ and most endocervical cultures went through a period of crisis when many cells died and a smaller subpopulation survived to become immortal. When experiments were performed using cultures attached to collagen-coated dishes, we observed less variability between immortalization efficiency of cells from ectocervix, endocervix and TZ (Fig 4D and S2 Table). To determine whether differences in immortalization efficiency were specific for HPV16, we performed assays using SV40 DNA (another small DNA tumor virus). Results showed that SV40 DNA caused immortalization of ectocervical cells > cells from TZ > cells from endocervix (Fig 4B). Thus, cells cultured from TZ were not more susceptible to immortalization with the complete genomes of HPV16, HPV18, or SV40.

### Immortalization by retroviruses encoding HPV16 *E6* and *E7*

**Fig 5:**
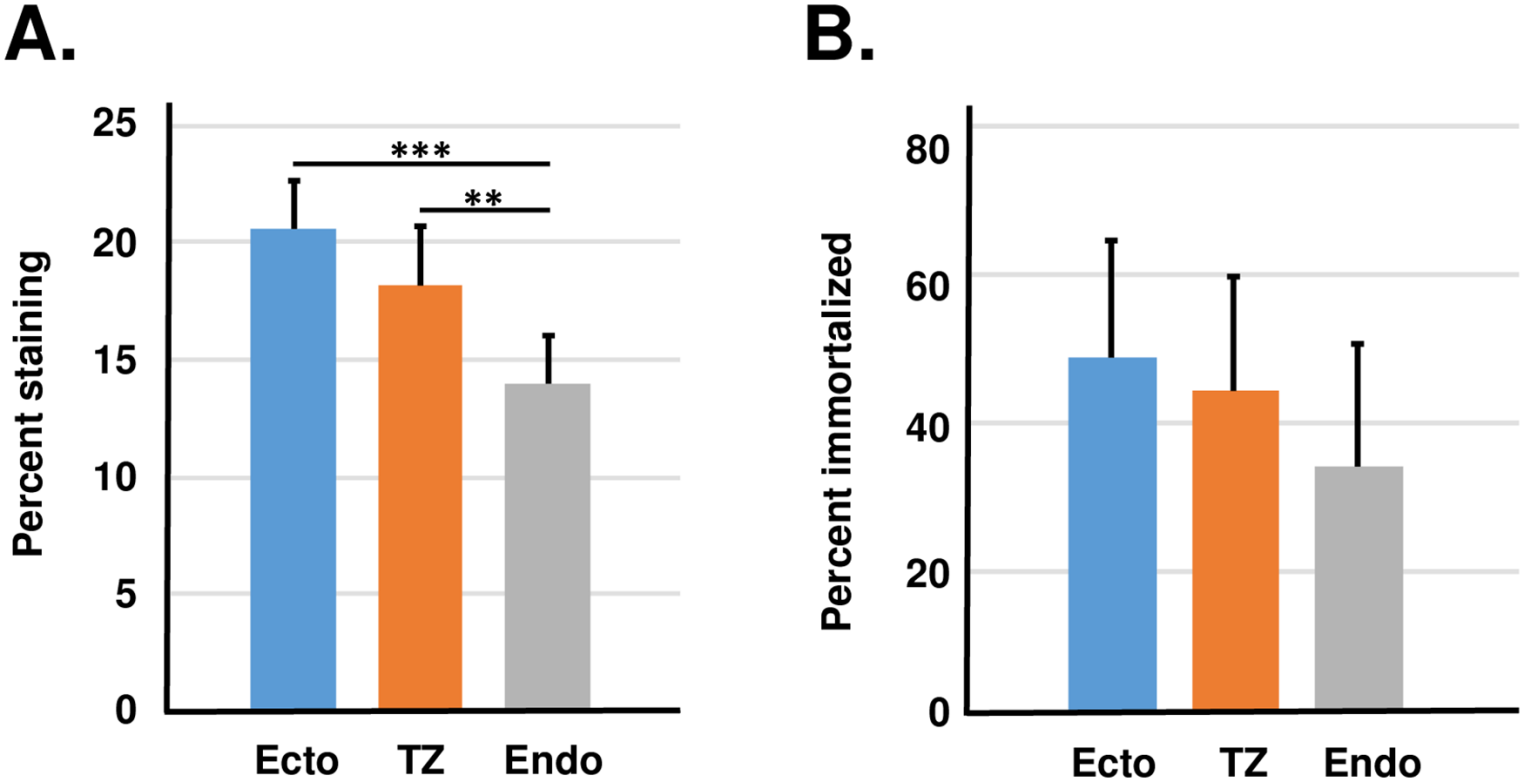
Infection and immortalization by retroviruses encoding HPV16 *E6*/*E7*. A. Infection efficiency of cells from ectocervix, TZ and endocervix. The bars represent the mean ± standard error of eight experiments using samples from different donors. B. Immortalization efficiency of cervical cells from each region that was normalized for differences in infection rate. Bars represent the mean ± standard error of eight experiments using samples from different donors. The asterisks show statistical differences [** P < 0.01; *** P < 0.001].

We performed immortalization assays using high-titer retroviruses encoding HPV-16 *E6* and *E7* genes and the neomycin resistance gene [17]. We used retroviruses because they infect cultured cervical cells with high efficiency [18]. Retroviruses containing only the neomycin resistance gene (no HPV) served as the negative control for immortalization. We infected secondary cultures from ectocervix, endocervix or TZ with undiluted supernatants from producer cell lines and selected in G418 to kill non-infected cells. Cultures were subcultured continually until those infected with neo-only viruses became senescent (2 to 4 passages). In preliminary experiments, approximately 90% of the cells were infected with undiluted virus stocks, and this high-level infection led to 100% immortalization in all experiments regardless of cervical region of origin (data not shown). Therefore, we diluted virus supernatants 1:4 and repeated experiments to measure the efficiency of infection (Fig 5A) and the resultant immortalization (Fig 5B and S3 Table). The diluted retrovirus supernatants infected 15 to 20% of cells in all cultures, and ectocervical cells were infected at the highest efficiency. After normalization for infection efficiency, we found that cells from ectocervix were immortalized slightly more efficiently than cells from other regions, although these differences were not statistically significant (Fig 5B). Thus, cells cultured from TZ were not more susceptible to immortalization with the HPV16 E6 / E7 retroviruses.

### Immortalization in stromal cell co-cultures

**Fig 6:**
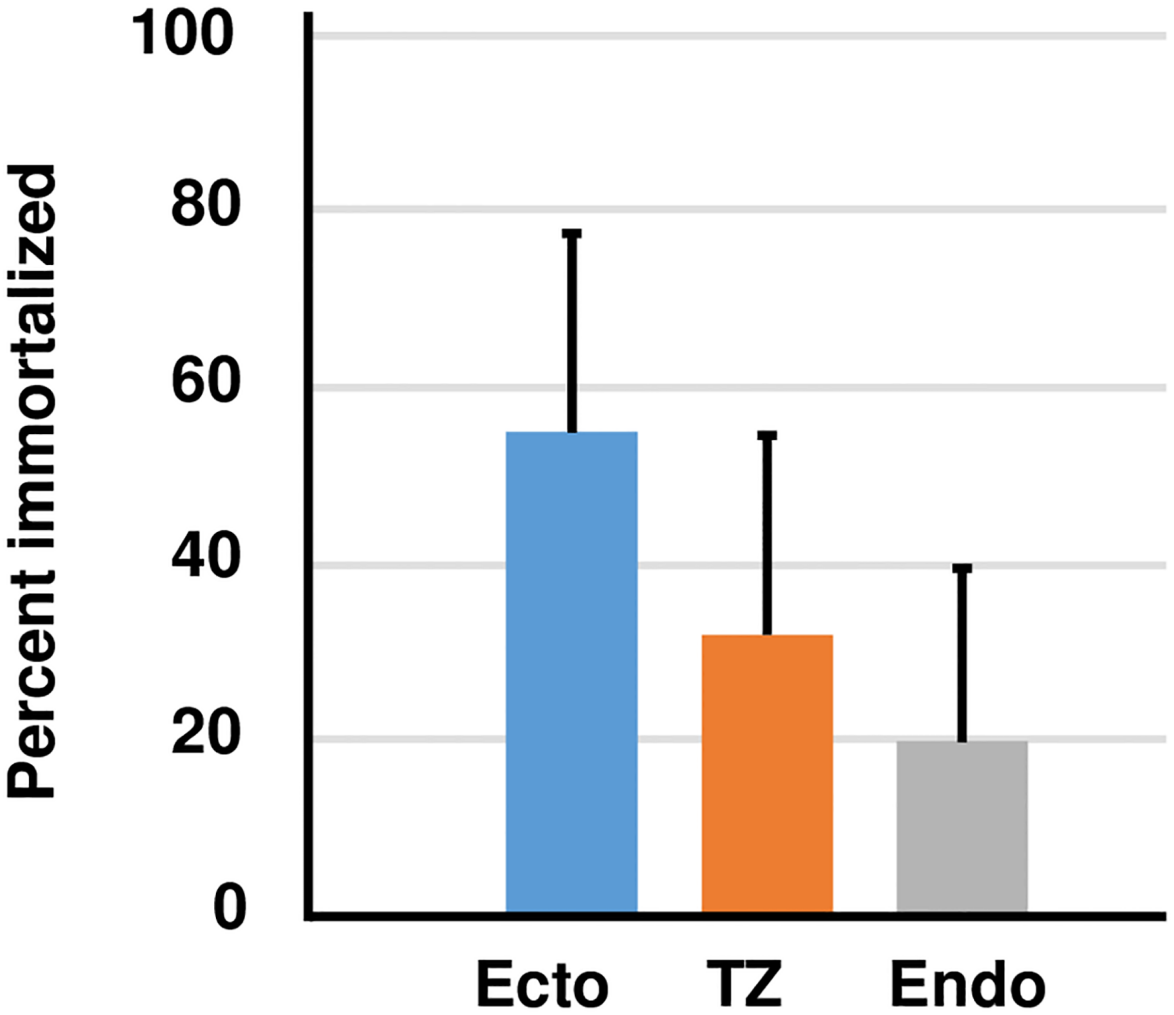
Immortalization by transfection with the complete HPV16 genome using co-cultures of epithelial and stromal cells. A. The bars represent the mean ± standard error of five experiments using samples from different donors. No significant difference was observed using ANOVA and Tukey Post-hoc test (SPSS), p > 0.05.

Keratinocytes can be co-cultured with stromal cells in serum-containing medium [14], and co-cultured keratinocytes behave differently in immortalization assays than cultures maintained in serum free medium [22]. Therefore, we examined the efficiency of immortalization by the complete HPV16 genome using co-cultures of primary human stromal cells and epithelial cells from ectocervix, endocervix and TZ (Fig 6 and S4 Table). We observed that the immortalization efficiency for each epithelial cell type in co-culture was similar to the efficiency observed in KSFM. Therefore, our results from three different immortalization assays (complete HPV16 genome, HPV16 E6/E7 retroviruses, or complete genome plus co-culture) showed that cells from the cervical TZ were not immortalized more efficiently than cells from ectocervix or endocervix.

### Development of HPV16 immortalized cell lines from TZ, ectocervix and endocervix

**Fig 7:**
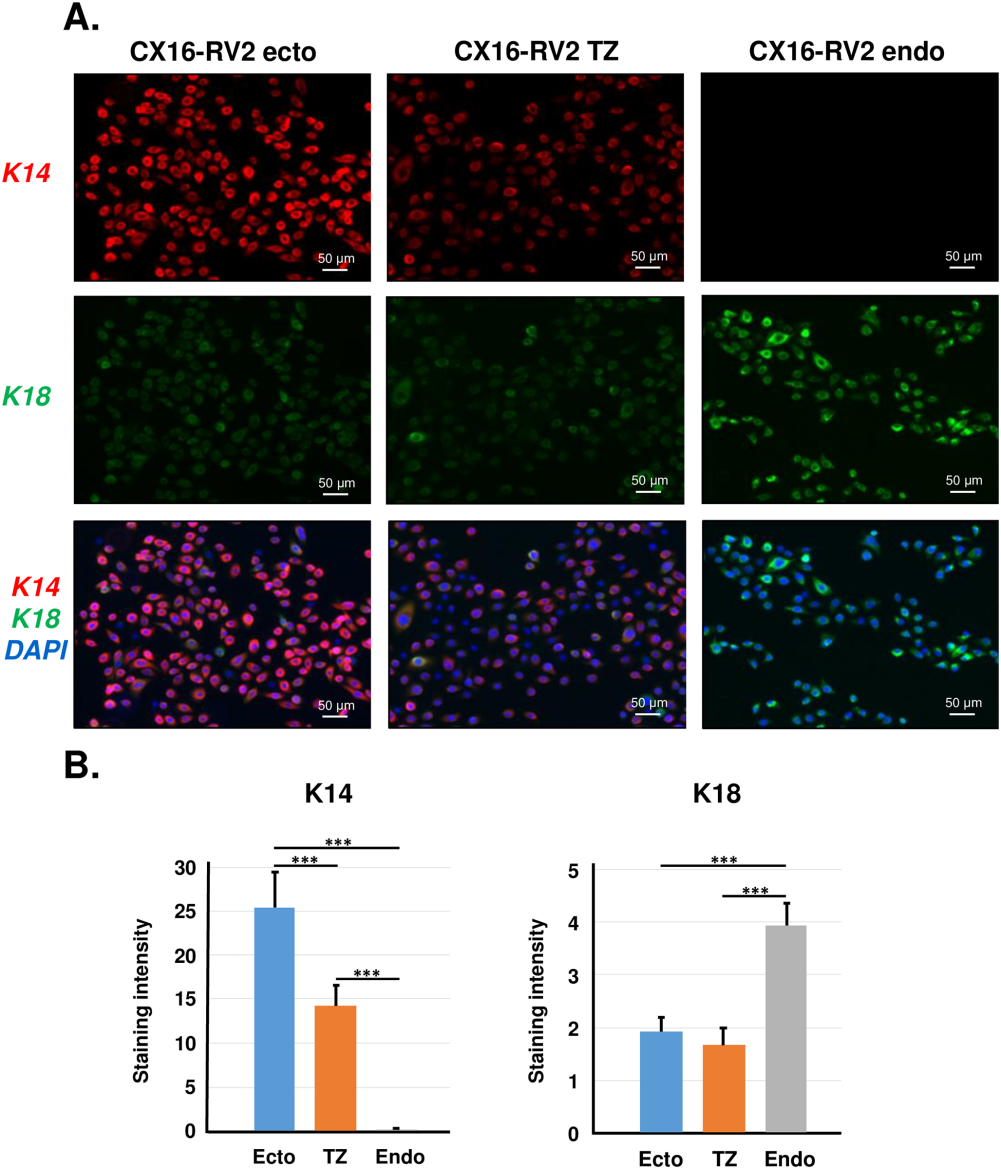
Keratin expression in HPV-immortalized cell lines. A. Immunofluorescence staining for K14 and K18 in TZ, ectocervix and endocervix-derived immortalized CX16-RV2 cell lines. B. Quantitative analysis of K14 and K18 staining intensity in 12 HPV16 immortalized cervical cell lines. Bars represent the mean ± standard error of four experiments using samples from different donors. The asterisks show statistical differences [*** P < 0.001].

HPV16-immortalized cells from ectocervix, endocervix and TZ grew continuously in culture and stable cell lines were derived (Table 1). We passed all of these cell lines at least 50 times and none showed evidence of slowing growth or senescence. We established matched cell lines from ectocervix, endocervix and TZ from eight different patients (24 individual cell lines). The origin of each cell line was authenticated by short tandem repeated sequence (STR) profiling performed by the American Type Culture Collection (S5 Table). STR results indicated that ectocervical, TZ and endocervical cell lines from each patient had the same STR pattern, confirming their common origin. STR results also showed that each of these 24 new cell lines differed in STR profile from all other established ATCC cell lines (i.e., there was no contamination with other existing cell lines). Table 1 shows several properties of 12 of these new cell lines from four different patients. HPV16-immortalized cell lines derived from endocervical cells usually went through a period of crisis when most cells died before a small subpopulation became immortal. Thus, it was more difficult to establish cell lines from endocervix. Most immortal cell lines resembled the primary cells from which they were derived with respect to keratin expression. Endocervical cell lines expressed high levels of K18 but never K14 (Fig 7). In contrast, ectocervical and TZ cell lines expressed abundant K14 but low levels of K18. One interesting feature of cell lines developed from TZ was their resistance to terminal differentiation in response to removal of growth factors (EGF and bovine pituitary extract) plus addition of 1.4 mM calcium to the culture medium. It will be important to characterize the differentiation of HPV16 immortalized cervical cell lines more completely either *in vivo* or in organotypic cultures. These newly established HPV16-immortalized cell lines are a unique tool to study cells from TZ, ectocervix and endocervix and compare their response to factors that promote cervical carcinogenesis.

**Table 1:** Properties of HPV16-immortalized cell lines.

Cell line	Region	Crisis^1^	K14^2^	K18	Diff.^3^
CX16-RV2	ecto	-	+++	+	+++
	TZ	-	++	+	+
	endo	+	-	+++	++
CX16-RV3	ecto	-	+++	++	+++
	TZ	-	+++	++	++
	endo	+	-	+++	+++
CX16-RV8	ecto	-	+++	++	+++
	TZ	-	+++	++	+
	endo	+	-	++	+++
CX16-RV9	ecto	-	+++	++	+++
	TZ	-	++	++	+
	endo	+	-	+++	++

^1^ Plus signs indicate that cells went through crisis before becoming immortalized.

^2^ Three + signs = high percentage of K14 positive cells, two + signs = moderate percent of K14 positive cells, and one + sign = low level of K14 positive cells.

^3^ Three + signs = well differentiated, two + signs = less differentiated, one + sign = poorly differentiated.

## Discussion

Cervical cancer is a major public health problem, and an important risk factor for this disease is persistent infection with high-risk HPV types, such as HPV16 [2]. Most infections with high-risk HPVs do not progress to cancer, and it is unclear why only a small subset undergo malignant conversion. An important observation is that over 90% of cervical cancers develop within the narrow region of the cervical TZ, even though HPV infects cells throughout the cervix [8]. We hypothesized that human epithelial cells cultured from TZ might be more susceptible to cellular immortalization, an early stage of cancer development. We used three different HPV16 immortalization assays to compare cells from ectocervix, endocervix and TZ. In contrast to our hypothesis, TZ cells were equally susceptible to immortalization as cells from other regions. Thus, our work suggests that other characteristics of the TZ contribute to the increased susceptibility to cervical carcinogenesis. We developed 24 HPV16-immortalized cell lines derived from TZ, ectocervix and endocervix of 8 different patients. These cell lines will be useful for investigating properties of TZ cells that make them susceptible to malignant development.

We confirmed that cells used in immortalization assays actually came from the respective regions of cervical tissue. Immunofluorescence staining showed that cultures derived from TZ, ectocervix or endocervix expressed the same pattern of K14 and K18 observed *in vivo*. Furthermore, cells cultured from TZ and endocervix expressed K17 and p63, markers for reserve cells that are the putative progenitors of cervical cancer [20]. Recent studies have described a discrete population of squamocolumnar junction cells (SC) that appear to be progenitors for cervical cancer [21]. To determine whether our immortalization assays included these SC cells, we examined expression of MMP7, a marker for SC cells *in vivo* [21]. We observed MMP7 expression in a small percentage of cells isolated from TZ, but we also detected MMP7 in cells cultured from ectocervix and endocervix. Previous work has shown that epidermal growth factor (EGF), which is present in KSFM, can induce MMP7 expression [23]. This might explain our observation that MMP7 was expressed in cells from all regions. Due to the influence of cell isolation and growth in culture, it might be difficult to relate expression of specific squamocolumnar junction markers *in vitro* to their pattern of expression within the cervix. Because SC cells represent a relatively small and discrete cell population, it is possible that only a few of these cells were present in cultures derived from TZ.

One potential concern was that cells from TZ and endocervix were difficult to transfect. We controlled for this by normalizing the immortalization efficiency (dividing by the transfection efficiency) or by using high-titer retroviruses to deliver HPV16 E6/E7 genes efficiently to all cells. Another concern was the significant variability in the immortalization efficiency of cells isolated from different patients (S1 Table). Therefore, we performed immortalization assays using a large number of tissues from multiple patients to confirm that our results were reproducible. An interesting observation was that HPV16-transduced cells (transfected or infected) from endocervix usually underwent crisis and most cells died or became senescent (Table 1). Thus, it was difficult to derive HPV-16-immortalized cell lines from endocervix.

Our results clearly showed that cells cultured from cervical TZ were not more susceptible to immortalization by HPV16. Thus, other characteristics of TZ may contribute to the increased susceptibility for malignant development. Transformation zones exist in epithelial tissues throughout the body and many are preferential sites for cancer development [11]. In cervical carcinogenesis, several characteristics of the TZ might be important [24]. Cells from TZ have been reported to be more susceptible to infection with HPV [25,26], possibly due to decreased immune responsiveness in this region [9]. Others have reported increased levels of estrogen receptors [10] or increased estrogen responsiveness in the TZ of a mouse model [27]. Thus, it is reasonable that multiple factors contribute to increased susceptibility of TZ to HPV-induced carcinogenesis.

We derived 24 HPV16-immortalized cell lines that will be useful for exploring differences that between TZ, ectocervix and endocervix. These matched ectocervical, endocervical and TZ lines came from eight different patients. We screened each cell line by PCR and confirmed that each expressed HPV16 E6 and E7 RNAs (data not shown). These new cell lines were unique by STR analysis because they did not match any previously known lines from ATCC (S5 Table). In seven of eight patients, endocervical, ectocervical and TZ lines had identical STR profiles, as expected. However, the endocervical cell line CX16-RV3 lost two markers on one chromosome relative to the ectocervical and TZ counterparts. This might be due to the extended period of crisis that occurs during immortalization of endocervical cells (Table 1). All of the HPV16-immortalized endocervical cell lines resembled primary cell cultures from endocervix as they expressed K18 but no K14. Immortalized cell lines from ectocervix and TZ resembled primary cultures since they expressed high levels of K14. However, some ectocervical and TZ cell lines also expressed low levels of K18, unlike their counterparts in primary culture.

This is consistent with previous work that showed cell immortalization induced expression of K18 in ectocervical cells [15].

Overall, we showed that epithelial cells from TZ do not have a higher susceptibility to immortalization by HPV16 than cells from ectocervix or endocervix. Thus, other properties of the TZ are likely to account for increased malignant progression. Another important outcome was the development of HPV16 immortalized cell lines from TZ, ectocervix and endocervix. These lines can help to examine cell properties that contribute to malignant progression.

## Major conclusions

1. Epithelial cells cultured from human TZ were not more susceptible to immortalization by HPV16 than cells from ectocervix or endocervix
2. Matched cell lines from ectocervix, endocervix and TZ were developed from 8 different patients (24 individual cell lines) and authenticated by STR profiling

## Supporting information

S1 Table Raw data of immortalization efficiency with the complete HPV16 genome. (TIF)

S2 Table Raw data of immortalization efficiency with HPV16 transfection and collagen-coated plates. (TIF)

S3 Table Raw data of immortalization efficiency with HPV16 E6/E7 retroviruses. (TIF)

S4 Table Raw data of immortalization efficiency with HPV16 transfection and co-culture. (TIF)

S5 Table Short tandem repeat (STR) profiling of HPV-immortalized cervical cell lines. The STR profile differed in cell lines derived from different patients, as expected. In contrast, the STR profile was identical in ecto-, endo-, and TZ-derived cell lines from all but one patient (data not shown). In CX16-RV3, the endo-cervical-derived line lost TH01 and D13S317 markers on one chromosome.
(TIF)
