# Supplementary figures and images for: Susceptibility of epithelial cells cultured from different regions of human cervix to HPV16-induced immortalization
Source: PLoS One. 2018 Jun 26;13(6):e0199761. doi: 10.1371/journal.pone.0199761 (PMC6019754; doi:10.1371/journal.pone.0199761)

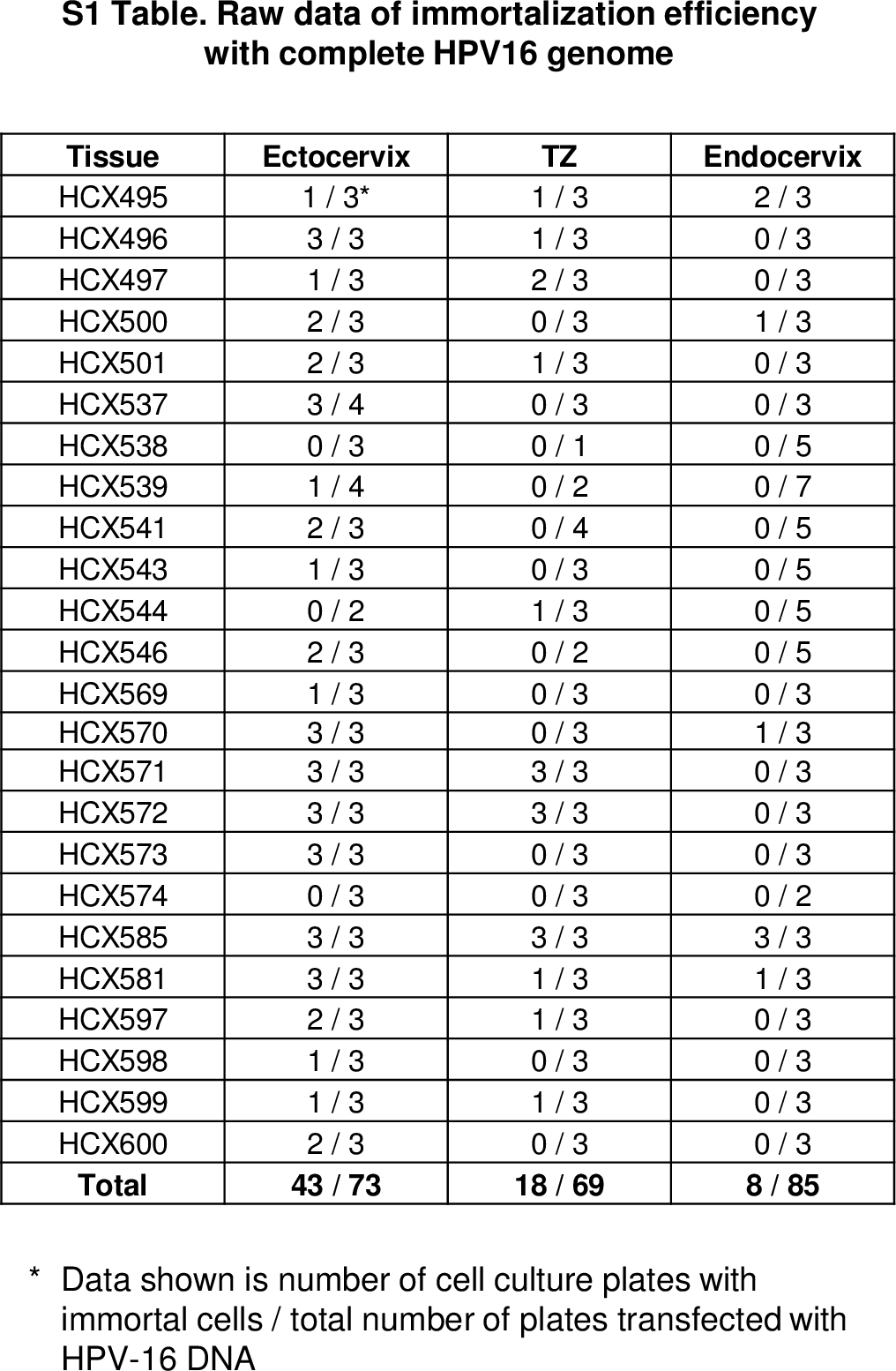

Supplement: S1 Table — (TIF) [file pone.0199761.s001.tif]

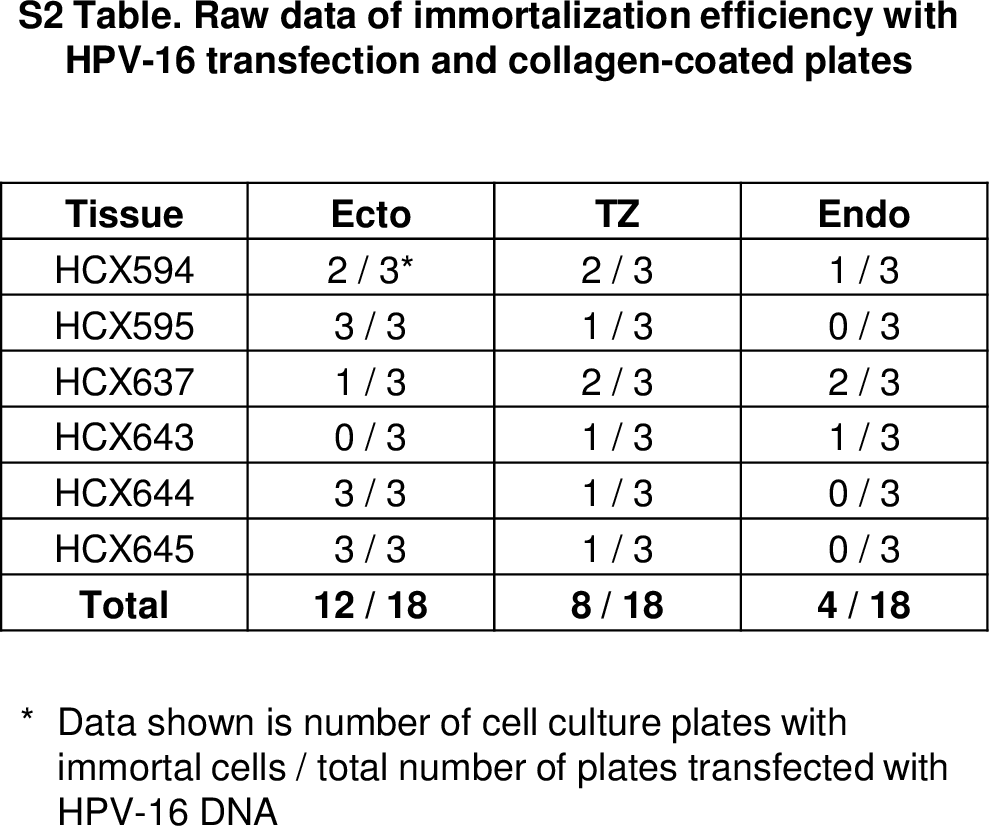

Supplement: S2 Table — (TIF) [file pone.0199761.s002.tif]

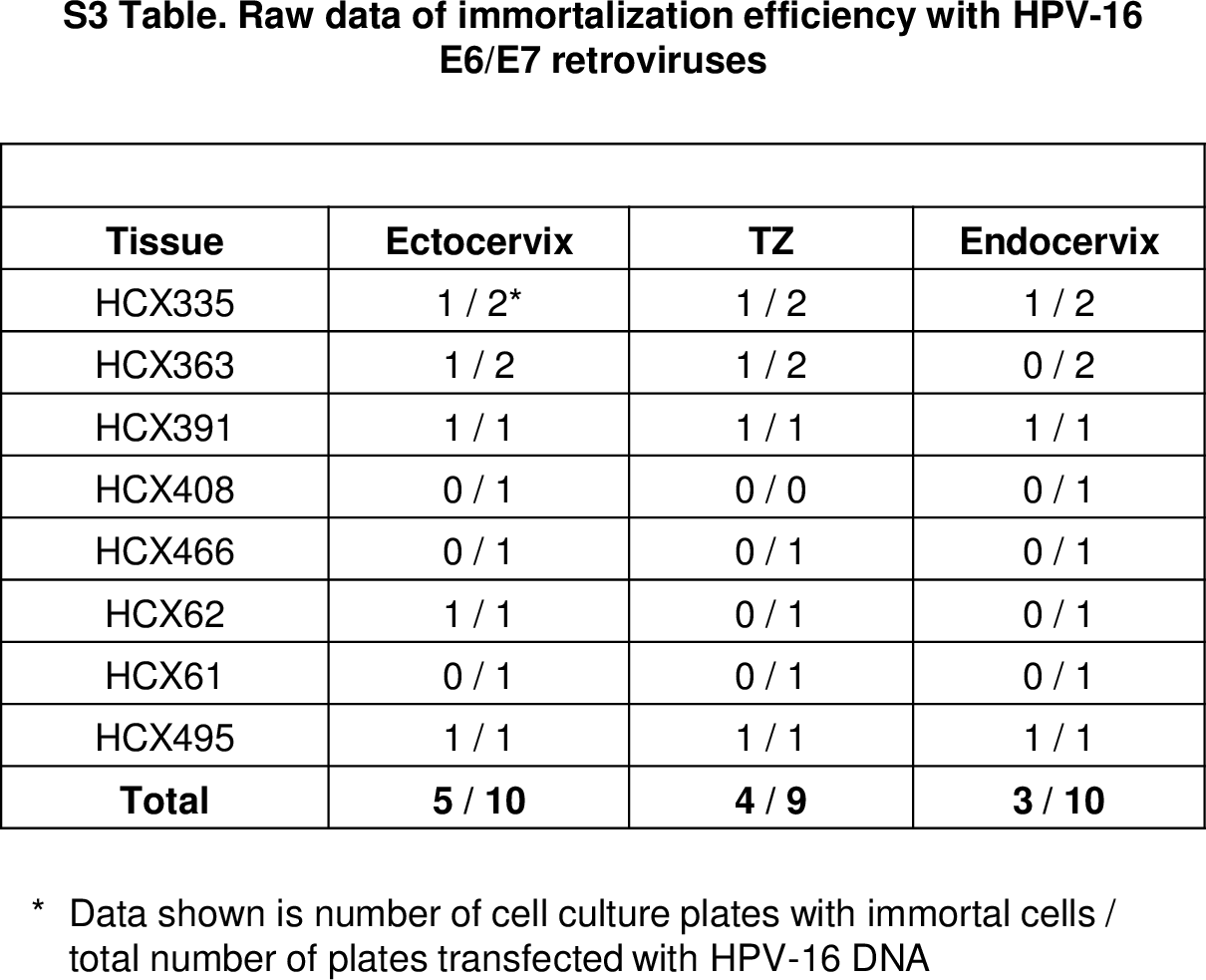

Supplement: S3 Table — (TIF) [file pone.0199761.s003.tif]

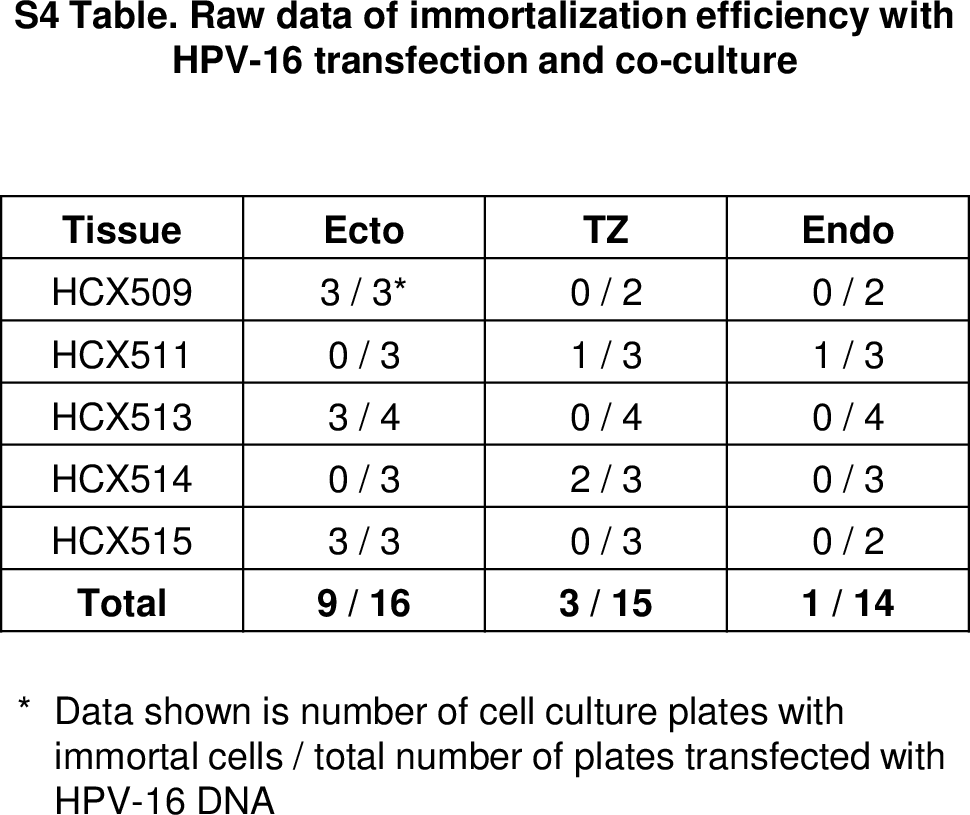

Supplement: S4 Table — (TIF) [file pone.0199761.s004.tif]

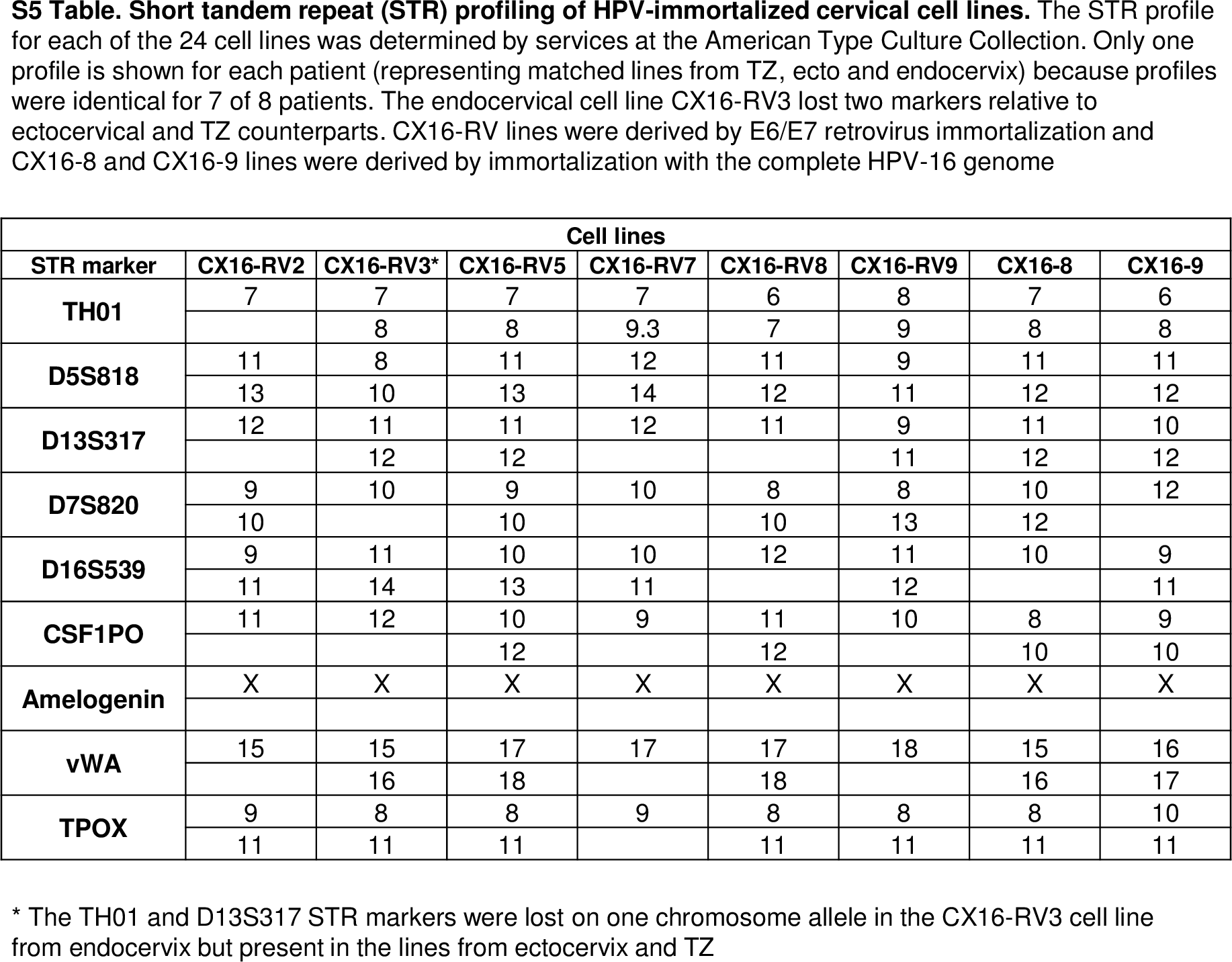

Supplement: S5 Table — The STR profile differed in cell lines derived from different patients, as expected. In contrast, the STR profile was identical in ecto-, endo-, and TZ-derived cell lines from all but one patient (data not shown). In CX16-RV3, the endo-cervical-derived line lost TH01 and D13S317 markers on one chromosome. (TIF) [file pone.0199761.s005.tif]
